# Comparing Approaches to Measuring Prospective Fertility Desires in the United States: A Population-based Study

**DOI:** 10.1007/s10995-026-04327-3

**Published:** 2026-10-01

**Authors:** Brooke W. Bullington, Isabel Muñoz, Corinne H. Rocca

**Affiliations:** 1 Division of Family Planning, Department of Obstetrics and Gynecology, University of Utah, Salt Lake City, USA; 2 Department of Obstetrics, Gynecology and Reproductive Sciences, Advancing New Standards in Reproductive Health (ANSIRH), School of Medicine, University of California, 1330 Broadway, Suite 1100, Oakland, San Francisco, CA 94612 USA

**Keywords:** Fertility desires, Pregnancy preferences, Measurement, Reproductive health, Desire to Avoid Pregnancy scale

## Abstract

**Objectives:**

Fertility desires are central to reproductive health, yet researchers lack consensus on how to best measure them. We compared participants’ responses to three individual questions – Timing (“How do you feel about having a child now or sometime in the future?”), Importance (“How important is it to you to avoid becoming pregnant now?”), and Wantedness (“Do you want to become pregnant in the next year?”) – to responses to the 14-item Desire to Avoid Pregnancy (DAP) scale, a psychometrically valid measure of pregnancy preferences.

**Methods:**

We analyzed data from the Surveys of Women, population-based longitudinal studies of reproductive-aged females in nine US states. We used multinomial logistic regression to generate predicted responses to each individual question by DAP score, and logistic regression to estimate annual incident pregnancy for each approach.

**Results:**

While responses to individual fertility desire questions aligned with DAP scores, the approaches yielded substantially different categorizations. The lowest and highest categories of Timing, Importance, and Wantedness largely captured individuals with low and high DAP scores, respectively. However, the highest categories also included large proportions of individuals with a moderate preference to avoid pregnancy (Timing: 45% of those with mid-range DAP scores, Importance: 43%, Wantedness: 70%). Similarly, the non-polar categories of the individual fertility desire questions included individuals across wide ranges of DAP scores.

**Conclusions for Practice:**

Asking about wanting pregnancy can identify those most open to pregnancy but does not differentiate fertility preferences among individuals not explicitly desiring pregnancy, which is most people. While single questions are efficient, lack of measurement precision or evidence that they accurately categorize respondents is a compelling drawback to their use.

## Significance

What Is Already Known on This Subject

What This Study Adds?

Researchers lack consensus on how to conceptualize fertility desires and use various measurement approaches to prospectively capture the construct.

We directly compared responses to three individual fertility desire questions with those from a formally developed, 14-item instrument, the Desire to Avoid Pregnancy (DAP) scale, using population-based data. Even though the individual questions identified many of the people most open to becoming pregnant soon, they did little to differentiate among those who did not explicitly desire pregnancy. Individual questions are more efficient but lack precision and may yield inaccurate categorizations.

## Introduction

Fertility desires and preferences are a central construct for scientific research, policy decision-making, and clinical care (Gipson et al., 2008; Santelli et al., 2003). Preferences influence behaviors, like contraceptive use and preconception practices, and offer important insight into reproductive health needs at the individual and population level (Chuang et al., 2010; Rocca et al., 2019, 2022). Accurately understanding the extent to which individuals desire pregnancy and childbearing is therefore critical to informing reproductive health interventions, guiding person-centered care, and tracking the degree to which people attain their preferences and achieve reproductive autonomy (Rocca et al., 2024).

Despite the importance of prospective fertility desires, reproductive health researchers and demographers lack consensus on how to conceptualize, define, and measure this construct. Scholars largely recognize that people have a diversity of feelings about pregnancy, and that uncertainty and ambiguity are important to capture (Bachrach & Newcomer, 1999; Kost & Zolna, 2019; Mumford et al., 2016; Petersen & Moos, 1997; Rocca et al., 2019; Santelli et al., 2003). Still, the lack of consensus has led researchers to develop and apply a range of prospective fertility desires questions across efforts in family planning and demographic research. Some questions, like the prospective One Key Question® (“Would you like to become pregnant in the next year?”), were designed to initiate contraceptive and reproductive health counseling in clinical settings (*One Key Question® Online 2025 | Power to Decide*, n.d.). Others are single questions created by researchers to assess different aspects of fertility desires, including how someone feels about having a child now or in the future (*Behavioral Risk Factor Surveillance System*, n.d.) or in the next month (Geist et al., 2019), how important it is for them to avoid pregnancy (Guzzo et al., 2025; Kaye et al., 2009), how much they want to get pregnant or avoid getting pregnant in the next month (Miller et al., 2013; Moreau et al., 2012), and future pregnancy plans (*Add Health*, n.d.; Geist et al., 2019). These questions have been utilized on large-scale surveys and studies, including Behavioral Risk Factor Surveillance System, AddHealth, Relationship Dynamics and Social Life Study, and HER Salt Lake, but have not undergone psychometric evaluation.

The Desire to Avoid Pregnancy (DAP) scale is the only multi-item, prospective measure of pregnancy and childbearing preferences that has been formally developed and tested psychometrically (Rocca et al., 2019). The DAP scale offers several advantages over other measurement approaches: the items were developed directly based on qualitative research documenting how women actually feel about experiencing a potential pregnancy. The scale’s items cover three conceptual domains (cognitive, affective, life consequences) and acknowledge that preferences change with changing life circumstances by referring specifically to preferences in the near-term (pregnancy in the next three months and childbearing within the next year). One limitation, however, is the scale’s length; with 14 items, survey completion time increases, which could contribute to respondent burden. It is therefore important to assess whether the DAP scale’s comprehensiveness justifies its length, and what is gained or lost when researchers use shorter, simpler survey questions.

In this study, we compared directly people’s responses to three different survey questions capturing fertility desires to their DAP scale scores using data from a large-scale, population-based, longitudinal study. We also compared the probability of annual incident pregnancy among individuals by responses to the questions or measures. Findings are important for evaluating the relative strengths and weaknesses of each measurement approach, determining which facets of fertility desires each approach may capture differently, and facilitating comparisons across studies that employ different measurement approaches.

## Methods

### Data Source

We used data from the Surveys of Women (SoW), longitudinal, population-based studies conducted between 2016–2023 across nine US states: Alabama (AL), Arizona (AZ), Delaware (DE), Iowa (IA), Maryland (MD), New Jersey (NJ), Ohio (OH), South Carolina (SC), and Wisconsin (WI). The surveys were initially designed to evaluate state-level contraceptive access initiatives and later expanded to examine broader reproductive health policy changes. Survey design and methodology have previously been published (Poland et al., 2023). Survey waves were staggered by state; for example, wave 1 (baseline) was in 2016 in DE and MD and in 2020 in AZ, NJ, and WI.

Data collection and management were conducted by NORC at the University of Chicago. Baseline survey samples were drawn from the USPS computerized delivery sequence file. The sampling strategy intentionally oversampled census tracts with greater proportions of non-white residents and lower population density. NORC employed a multi-mode data collection strategy, including initial mailings and follow-up for nonrespondents. Surveys were completed via web-based or paper self-administered questionnaires.

Participants were eligible if they were aged 18–44 years. In AL, DE, IA, MD, OH, and SC, respondents had to self-identify as female to be eligible; in AZ, NJ, and WI, respondents could identify as female, transgender, gender expansive, or “something else.” Each state’s surveys were comprised of up to five survey waves: a baseline (wave 1) survey completed by a representative sample of reproductive-aged females, two or three follow-up surveys of wave 1 participants (waves 2–4), and a cross-sectional endline comprised of a new representative sample (wave 5). Follow-up surveys were administered every one to two years.

### Measures

We assessed four distinct approaches to capturing different aspects of fertility desires:

#### Timing

Across all states at all waves, participants were asked, “How do you feel about having a child now or sometime in the future?” with response options referring to desired timing of having a child (<12 months, 12 months-<2 years, 2-<5 years, ≥5 years, or don’t know) or don’t want a child.

#### Importance

Across all states at all waves, participants were asked, “How important is it to you to avoid becoming pregnant now?” with response options on a five-point Likert scale ranging from “not important at all” to “very important.”

#### Wantedness

At wave 1 in IA, OH, SC, and AL and wave 5 in SC and AL, participants were asked a modified version of One Key Question®, either: “Overall, thinking about the next year, do you want to become pregnant?” (in SC and AL) or “Do you want to become pregnant in the next year?” (in OH and IA). Response options varied slightly by state: In SC and AL, response options were: “Yes,” “No,” “Unsure,” and “Don’t care”; in IA and OH, they were: “Yes,” “No,” “OK either way,” and “Don’t care.” For analyses, we collapsed “Unsure,” “OK either way,” and “Don’t care.” We were unable to examine these categories separately due to variation in how they were presented among surveys and states.

#### Desire to Avoid Pregnancy (DAP) Scale

Across all states and waves except for baseline in DE and MD, participants completed the DAP scale (Rocca et al., 2019). Developed based on preference construction theory, the DAP scale is designed explicitly to accommodate ambiguity and uncertainty, acknowledging that many individuals do not hold clear intentions regarding future pregnancy, better aligning with many people’s lived experiences (Bhrolcháin & Beaujouan, 2019; Kendall et al., 2005). The scale is made up of 14 items that assess respondents’ feelings about a potential pregnancy (“thinking about becoming pregnant in the next three months makes me feel unhappy”) and, correspondingly, having and raising a child (“If I had a baby in the next year, it would be hard for me to manage raising the child”). Participants respond to each item on a five-point Likert scale (strongly agree to strongly disagree). Scores range from 0 to 4 (0: greater openness to pregnancy; 4: stronger preference to avoid pregnancy), and were categorized (low DAP: ≤1.5, mid-range DAP: >1.5 to ≤2.5, high DAP: >2.5). We used established DAP groupings in some analyses to facilitate presentation and interpretation (Rocca et al., 2025). The scale has demonstrated high internal consistency and internal and external structure validity (Hall et al., 2022; Rocca et al., 2019).

### Analysis

Analyses comparing fertility desires measurement included data collected at waves 1 and 5, or two cross-sectional samples from seven states and one (wave 5) from DE and MD. The DAP scale was not yet available for use in 2016, when the DE/MD wave 1 data collection occurred. We did not include fertility desire data from waves 2–4 due to inconsistencies in how survey questions were administered over time and between states. We do not intend for results to generalize to a particular state or year, so we combined data across waves 1 and 5 for analyses. Our total analytic sample included 32,802 unique participants, including 27,138 for analyses of Timing, 29,792 for analyses of Importance, and 10,969 for analyses of Wantedness, which was only administered in a subset of states at waves 1 and 5. Analyses examining incident pregnancy excluded wave 5 due to the need for longitudinal data; for these analyses, we restricted to wave 1, determining incident pregnancy at wave 2.

We described the distribution of responses to each individual fertility desire question (Timing, Importance, and Wantedness) within each DAP score category (low, mid-range, high). We used multinomial logistic regression models to generate predicted probabilities of selecting each response option as a function of continuous DAP score and graphed these probabilities. We also described the distribution of each DAP score category within each individual fertility desire question.

To investigate sociodemographic factors associated with “divergent” responses among measurement approaches, we used logistic regression to derive predicted probabilities of responding with the lowest degree of desire (e.g.,“No” to Wantedness) among participants with low DAP scores by characteristic. We examined characteristics that have previously been found to be associated with pregnancy preferences (Bullington et al., 2025; Samari et al., 2020). We intended to examine the inverse (e.g., “Yes” to Wantedness among those with high DAP scores), but too few responses were divergent in this way.

To model the association between responses to each fertility desire question and reported incident pregnancy the following year, we fit a series of logistic regression models. We present predicted probabilities of incident pregnancy and 95% confidence intervals based on these models. For this portion of the analysis, we limited our sample to: 1) participants who could experience pregnancy (not pregnant, had not had a tubal ligation, not infertile) at wave 1, and 2) had complete data on incident pregnancy at wave 2. Because each fertility desire question was administered differently in different states/waves, Ns included in each model varied.

All models accounted for state as a fixed effect and employed survey weights, assigned by NORC for each wave of data in each state using a five-step weighting process that included base weights, adjustment of unknown eligibility, non-response, and household size, and poststratification. For wave 2, additional weights were applied to account for loss to follow-up from wave 1 (Poland et al., 2023).

Deidentified data for this study were provided to the research team by NORC for these secondary data analyses. As such the research was classified as Category 4 exempt by the University of California, San Francisco Institutional Review Board.

## Results

The combined sample of participants at waves 1 and 5 were of all reproductive ages (Table 1). Just over half (52%) were nulliparous, and 7% had given birth in the prior year. Most lived with a romantic partner (61%), had some college education (81%), and were employed (74%). About 5% were Asian non-Hispanic, 15% Black non-Hispanic, 4% Hispanic, 13% Multiracial or another race non-Hispanic, and 63% white non-Hispanic. Most participants (92%) reported they were in excellent, very good, or good health.

**Table 1:** Characteristics and fertility desires of reproductive-aged females from nine US states in the Surveys of Women^1^ (unweighted N=32,802)

	*Unweighted N*	*Weighted %*
*Age group in years (N=32,802)*		
18–24	4712	25.1%
25–29	5389	20.0%
30–34	7052	21.7%
35–39	7351	15.9%
40–44	8298	17.3%
*Parity (N=31,710)*		
Nulliparous (0)	13,589	52.4%
Primiparous (1)	5532	16.3%
Multiparous (2)	7531	18.4%
Multiparous (3 +)	5058	13.0%
*Prior year birth (N=31,646)*		
Yes	2297	7.3%
No	29,349	92.7%
*Lives with romantic partner (N=32,456)*		
Yes	21,920	60.9%
No	10,536	39.1%
*Highest level of education (N=32,802)*		
High school/equivalent or less	4662	19.0%
Some college	10,541	41.5%
Bachelor’s degree or more	17,599	39.5%
*Employment (N=32,802)*		
Employed	25,587	74.3%
Unemployed	1805	6.8%
Out of work force	5410	19.0%
*Race & ethnicity (N=32,788)*		
Asian, non-Hispanic	1264	5.0%
Black, non-Hispanic	4047	15.0%
Hispanic	1009	4.3%
Multiracial or another race, non-Hispanic	3145	13.0%
White, non-Hispanic	23,323	62.8%
*Importance of religion in life (religiosity) (N=26,480)*		
Very important	9834	32.8%
Somewhat important	7939	31.3%
Not important	8707	35.9%
*Self-rated health (N=32,755)*		
Excellent, very good, or good	30,302	92.1%
Fair or poor	2453	7.9%
Desire to Avoid Pregnancy (DAP) Scale score, mean (SD) (N=32,802)	2.4	1.1
Desire to Avoid Pregnancy (DAP) Scale score, median (IQR) (N=32,802)	2.5	1.7, 3.4
Desire to Avoid Pregnancy (DAP) Scale score category (N=32,802)		
Low DAP	6907	19.9%
Mid-range DAP	9882	28.8%
High DAP	16,013	51.3%
*Timing* How do you feel about having a child now or sometime in the future?^2^ (N=27,138)		
I do want to have one, less than 1 year from now	2315	8.0%
I do want to have one, between 1 year and less than 2 years from now	3075	11.8%
I do want to have one, between 2 years and less than 5 years from now	3330	14.9%
I do want to have one, 5 or more years from now	1775	10.2%
I do want to have one, but I’m not sure when	3027	12.6%
I don’t want to have one	13,616	42.5%
*Importance* How important is it to you to avoid becoming pregnant now?^2^ (N=29,792)		
Not important at all	3892	12.6%
Somewhat unimportant	744	2.5%
Neither important nor unimportant	3232	10.6%
Somewhat important	4341	13.6%
Very important	17,583	60.6%
*Wantedness* *Overall, thinking about the next year, do you want to become pregnant?* *or* Do you want to become pregnant in the next year?^3^ (N=10,969)		
Yes	1374	12.5%
Unsure, OK either way, or don’t care	1762	16.8%
No	7833	70.8%

^1^The sample includes all participants who either completed baseline (wave 1) or endline (wave 5) who were not missing the Desire to Avoid Pregnancy score.

^2^Measured in all states at waves 1 and 5

^3^Measured only at wave 1 in Iowa (IA), Ohio (OH), South Carolina (SC) and Alabama (AL) and wave 5 in SC and AL. Survey question wording varied between SC/AL and OH/IA

The median DAP score was 2.5 (interquartile range: 1.7, 3.4), and the mean was 2.4 (standard deviation: 1.1, Table 1). About 20% of participants had a low DAP score, 29% a mid-range DAP score, and 51% a high DAP score. When asked how they feel about having a child now or sometime in the future, 8% wanted a child within the year, 12% in 1-<2 years, 15% in 2-<5 years, 10% in 5 years or more, 13% wanted a child but were unsure about timing, and 43% did not want a child. When asked how important it is to them to avoid becoming pregnant now, 13% responded not important at all, 3% somewhat unimportant, 11% neither unimportant nor important, 14% somewhat important, and 61% very important. When asked if they want to become pregnant in the next year, 13% said yes, 17% said unsure, OK either way, or don’t care, and 71% said no.

### Comparing Measurement Approaches

Although participants’ responses to the individual pregnancy desire questions aligned roughly with their DAP score groupings, there was also substantial incongruence, particularly along the middle ranges of preference to avoid pregnancy.

#### Timing

About two-thirds of participants with low DAP scores responded they wanted a child in the next year (36%) or in 1-<2 years (33%) (Table 2). About three quarters of participants with high DAP scores did not want a child (57%) or wanted a in child 5+ years (16%). While 40% of those with mid-range DAP scores did not want a child, others did in 1-<2 years (17%), 2-<5 years (21%), or were unsure when (15%).

**Table 2:** Comparing categories of the Desire to Avoid Pregnancy Scale and other fertility desires questions

		Desire to Avoid Pregnancy Scale score					
		Column %			Row %		
		Low DAP	Mid-range DAP	High DAP	Low DAP	Mid- range DAP	High DAP
Timing: How do you feel about having a child now or sometime in the future?	Want, <1 year	36	3	<1	88	9	2
	Want, 1-<2 years	33	17	2	54	36	9
	Want, 2-<5 years	11	21	14	14	37	49
	Want, 5+ years	1	5	16	2	12	86
	Want, not sure when	14	15	11	22	30	48
	Don’t want	6	40	57	3	25	73
Importance: How important is it to you to avoid becoming pregnant now?	Not important	48	9	2	73	19	8
	Somewhat unimportant	7	4	<1	57	39	4
	Neither important nor unimportant	21	19	2	39	49	12
	Somewhat important	15	26	7	21	54	25
	Very important	9	43	89	3	20	77
Wantedness: Thinking about the next year, do you want to become pregnant? or Do you want to become pregnant in the next year?	Yes	53	3	<1	92	7	1
	Unsure, OK either way, or don’t care	36	27	2	47	47	5
	No	11	70	98	3	29	68

**Fig. 1:**
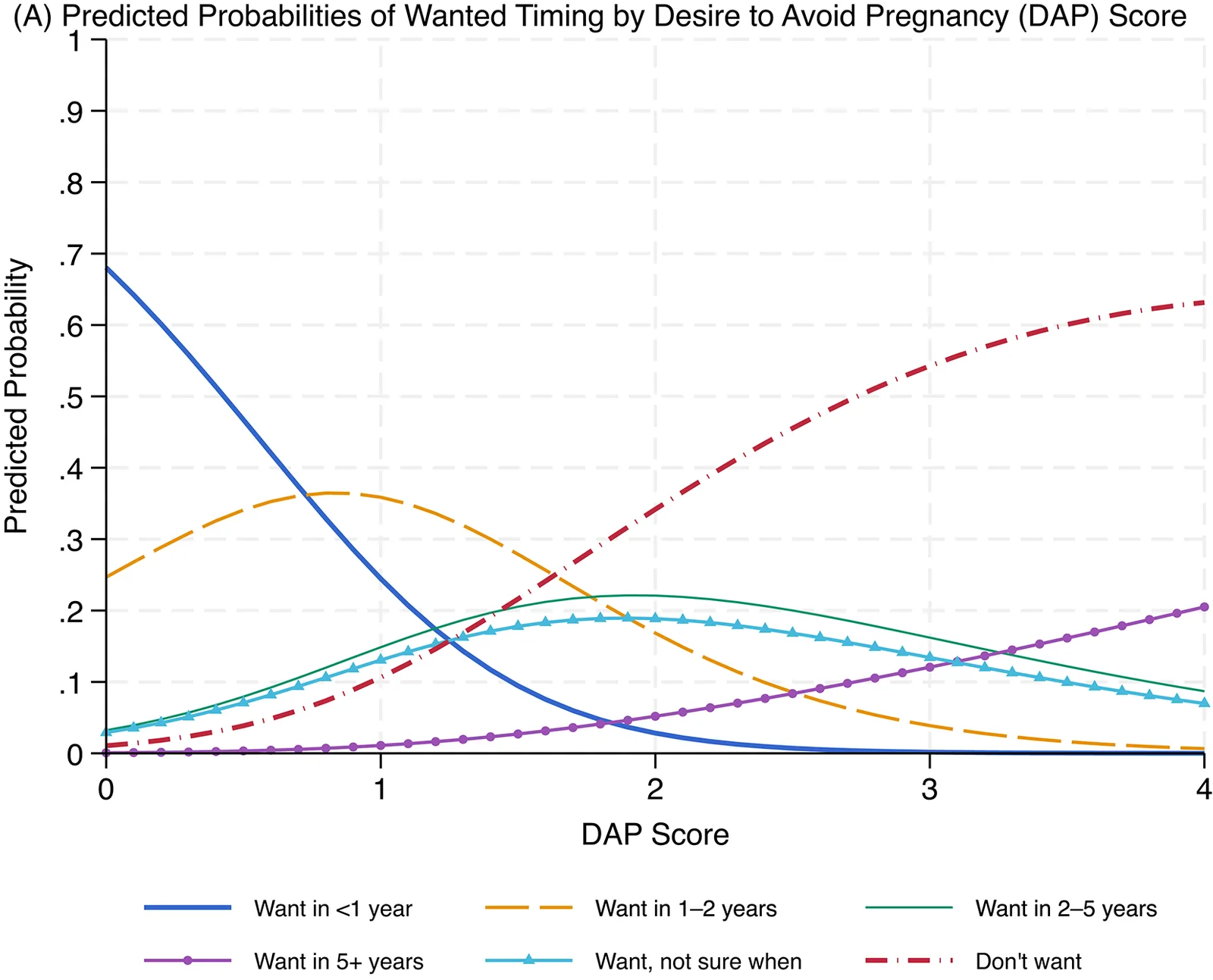

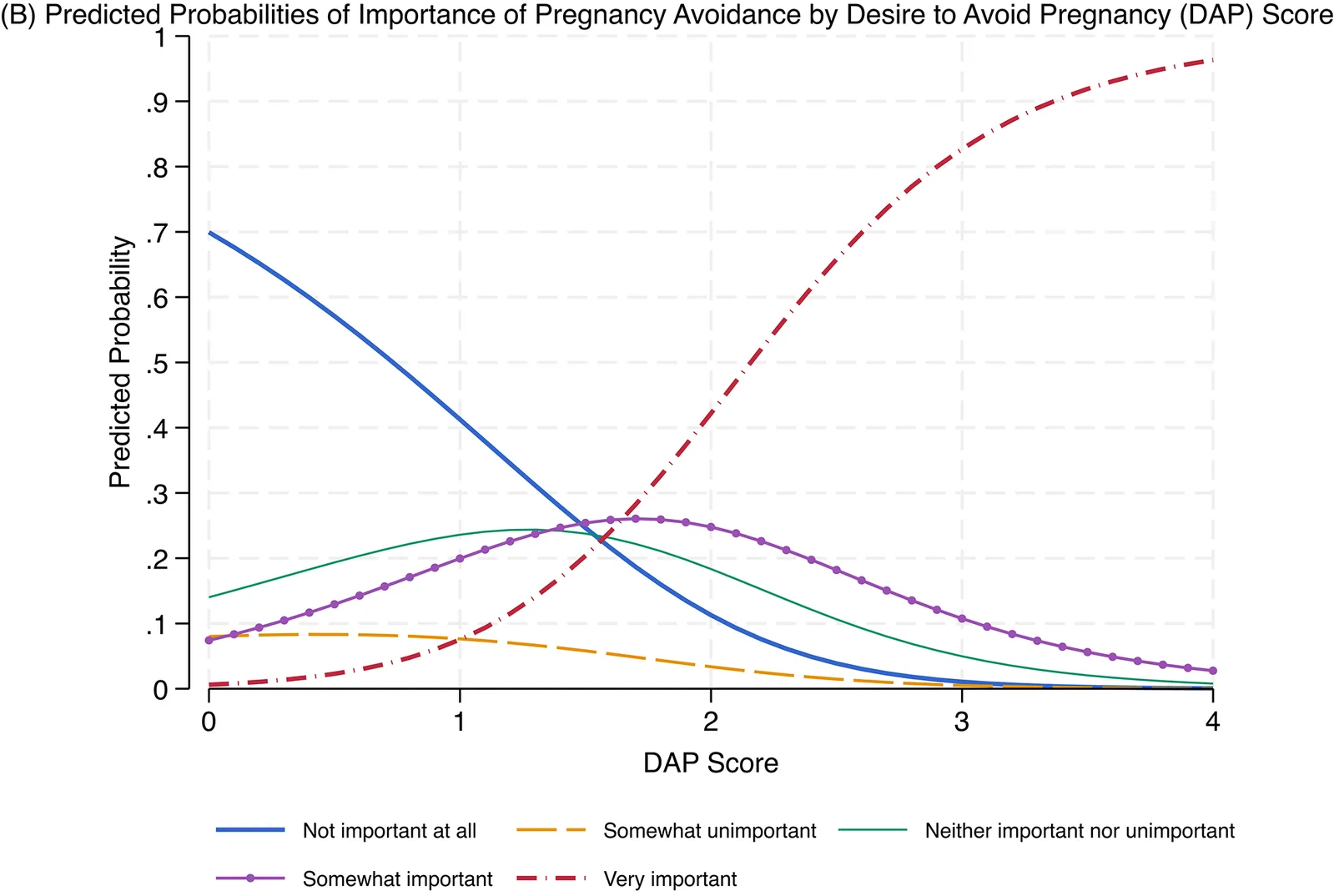

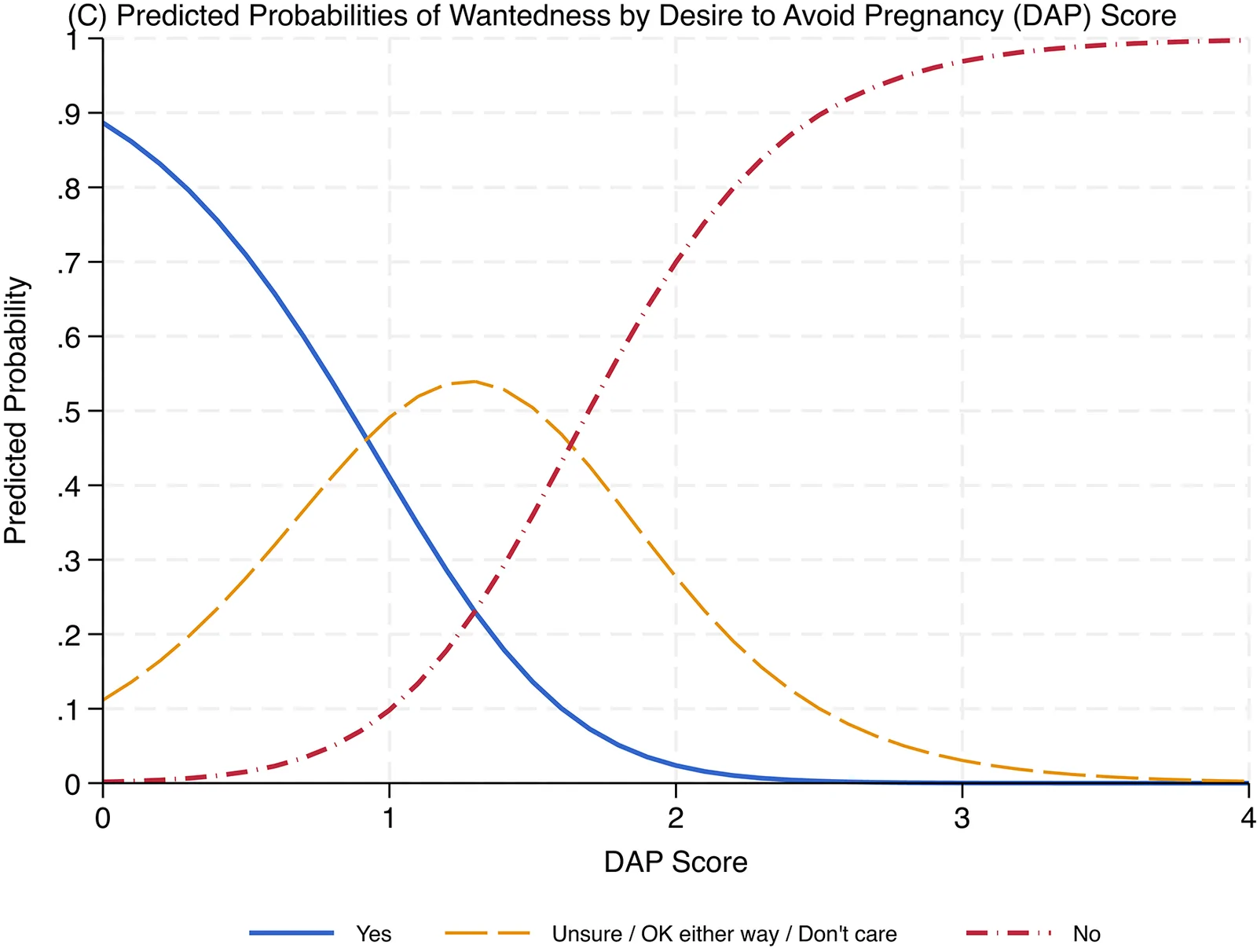
Predicted probabilities of fertility desire question response options by continuous Desire to Avoid Pregnancy Scale score

While DAP scores largely captured Timing, there was great diversity in Timing among participants in the mid to high ranges of DAP scores (Fig. 1A). For instance, participants along a broad range of DAP scores (roughly 1.7 and above) most commonly did not want a child ever. Similarly, participants with a very broad range of preferences about a pregnancy in the next three months (per the DAP scale) said they wanted a child in 2–5 years, 5+ years, or did not know when.

Similarly, 88% of participants who indicated wanting a child in the next year and 54% of those who wanted a child in 1-<2 years had low DAP scores, and 86% of those who indicated wanting a child in 5+ years and 73% of those never wanting a child had high DAP scores (Table 2). Those who wanted a child in 2-<5 years or who were unsure of desired timing had a wide range of DAP scores.

#### Importance

Similar patterns emerged between DAP scores and Importance: over half (55%) of participants with low DAP scores reported that avoiding pregnancy was not at all important or somewhat unimportant to them, and 89% with high DAP scores said it was very important (Table 2).

Most participants with mid-range DAP scores said avoiding pregnancy was very (43%) or somewhat (26%) important, and 19% said it was neither important nor unimportant. At all ranges of DAP scores, “not important at all” or “very important” were the most probable predicted responses (Fig. 1B). “Somewhat unimportant” was very improbable across all ranges of DAP scores; and “somewhat important” and “neither” were similarly improbable responses across DAP scores and had wide curves, reflecting that participants across a broad range of DAP scores (0–3) were similarly likely to endorse these responses.

Most participants who reported that avoiding pregnancy was not important at all to them had low DAP scores (73%), and most participants who indicated avoiding pregnancy was very important had high DAP scores (77%, Table 2). Those who reported that avoiding pregnancy was neither important nor unimportant or somewhat important had a range of DAP scores.

#### Wantedness

Responses to the Wantedness question were strongly related to DAP scores: 53% of those with low DAP scores wanted to become pregnant in the next year, while 36% were unsure, OK either way, or did not care (Table 2). Among those with high DAP scores, 98% did not want to become pregnant in the next year. The majority of participants with mid-range DAP scores did not want to get pregnant in the next year (70%), and 27% were unsure, OK either way, or did not care.

Examining predicted probabilities, “yes” was the most likely response among participants in the low DAP score range, and “no” was most likely among those with DAP scores >1.6 (Fig. 1C). “Unsure, OK either way, or don’t care” was probable (≥ 50% likelihood) among people with DAP scores between 1 and 1.5.

Most participants who indicated wanting to become pregnant in the next year (92%) had low DAP scores, and only 1% had high DAP scores (Table 2). However, only 68% of participants who reported not wanting to become pregnant in the next year had high DAP scores, and 29% had mid-range DAP scores. Nearly all participants who were unsure, OK either way, or did not care had low (47%) or mid-range (47%) DAP scores.

Examining sociodemographic characteristics associated with responding with the lowest degree of fertility desire among those with low DAP scores (the greatest openness to pregnancy), the predicted probability of responding “no” to next year Wantedness among those with low DAP scores was elevated among participants at the ends of the age range (aged 18–24 [16%] and 40–44 [22%] vs. 7% aged 30–34 years) (Table 3). Low DAP score/no Wantedness was also elevated among multiparous participants (21% for parity 2, 24% for parity 3) vs. nulliparous and primiparous participants (both 6%). It was also elevated among Hispanic participants (18%). Results were generally consistent across the two other individual questions, Timing and Importance (not shown).

**Table 3:** Predicted probability of responding “No” to Wantedness among those with low DAP scores by sociodemographic characteristics (unweighted N=2,128)

	Predicted probability of answering “no” to Wantedness	95% confidence interval
Overall	0.11	0.09, 0.13
*Age group in years*		
18–24	0.16	0.08, 0.24
25–29	0.08	0.04, 0.12
30–34	0.07	0.05, 0.09^a^
35–39	0.11	0.08, 0.15^b^
40–44	0.22	0.17, 0.27^c,d,e^
*Parity*		
Nulliparous (0)	0.06	0.04, 0.08
Primiparous (1)	0.06	0.04, 0.08
Multiparous (2)	0.21	0.16, 0.26^f,g^
Multiparous (3 +)	0.24	0.17, 0.31^ h,i^
*Prior year birth*		
Yes	0.13	0.07, 0.20
No	0.11	0.09, 0.12
*Lives with romantic partner*		
Yes	0.11	0.09, 0.13
No	0.09	0.05, 0.13
*Highest level of education*		
High school/equivalent or less	0.09	0.05, 0.13
Some college	0.10	0.08, 0.13
Bachelor’s degree or more	0.13	0.10, 0.16
*Employment*		
Employed	0.11	0.09, 0.13
Unemployed	0.15	0.04, 0.25
Out of work force	0.11	0.07, 0.14
*Race & ethnicity*		
Asian, non-Hispanic	0.08	0.00, 0.20
Black, non-Hispanic	0.07	0.03, 0.11
Hispanic	0.18	0.07, 0.29^j^
Multiracial or another race, non-Hispanic	0.06	0.02, 0.11^ k^
White, non-Hispanic	0.12	0.10, 0.14
*Importance of religion in life (religiosity)*		
Very important	0.11	0.09, 0.13
Somewhat important	0.11	0.07, 0.14
Not important	0.10	0.06, 0.15
*Self-rated health*		
Excellent, very good, or good	0.11	0.09, 0.13
Fair or poor	0.08	0.03, 0.14

^a^p<0.01 vs. 18–24; ^b^p<0.05 vs. 30–34; ^c^p<0.001 vs. 25–29; ^d^p<0.001 vs. 30–34; ^e^p<0.01 vs. 40–44

^f^p<0.001 vs. parity 0; ^g^p<0.001 vs. parity 1; ^h^p<0.001 vs. parity 0; ^I^p<0.001 vs. parity 1

^j^p<0.05 vs. Black non- Hispanic; ^k^ p<0.05 vs. Hispanic

### Predictive Ability of Pregnancy Among Measurement Approaches

Across all four approaches, incident pregnancy was associated with fertility desires (Table 4). Participants with low DAP scores, those who wanted a child within one year, those who reported that avoiding pregnancy was not important at all or somewhat unimportant, and those who said they wanted to become pregnant in the next year had the highest predicted probabilities of pregnancy (between 0.25 and 0.40). In contrast, those with high DAP scores, who did not want a child, who considered avoiding pregnancy very important, and who reported not wanting to become pregnant in the next year had the lowest predicted probabilities of pregnancy (between 0.02 and 0.05). Incident pregnancy among those in the middle categories of fertility desires questions fell between these values and were generally more similar to those who desired to avoid (vs. wanted) pregnancy.

**Table 4:** Predicted probability of incident pregnancy at wave 2 by different fertility desires measures at wave 1

	Predicted probability of incident pregnancy	95% confidence interval	p-value
Desire to Avoid Pregnancy scale (N=7,396)^1^			
*Categorical DAP scores*			
Low DAP	0.25	0.22, 0.29	ref
Mid-range DAP	0.09	0.07, 0.10	P<0.001 vs. low
High DAP	0.04	0.03, 0.05	P<0.001 vs. low P<0.001 vs. mid
*Continuous DAP scores*			
DAP=0	0.38	0.32, 0.44	ref
DAP=1	0.21	0.18, 0.23	P<0.001 vs. 0
DAP=2	0.10	0.09, 0.11	P<0.001 vs. 0 P<0.001 vs. 1
DAP=3	0.05	0.04, 0.05	P<0.001 vs. 0 P<0.001 vs. 1 P<0.001 vs. 2
DAP=4	0.02	0.01, 0.03	P<0.001 vs. 0 P<0.001 vs. 1 P<0.001 vs. 2 P<0.001 vs. 3
*Timing:* How do you feel about having a child now or sometime in the future? (N=8,034)^2^			
Want, < 1 year	0.40	0.34, 0.46	ref
Want, 1-<2 years	0.18	0.15, 0.22	p<0.001 vs. want, <1 yr
Want, 2-<5 years	0.13	0.09, 0.16	p<0.001 vs. want, <1 yr p=0.02 vs. want, 1-<2yrs
Want, 5+ years	0.04	0.01, 0.06	p<0.001 vs. want, <1 yr p<0.001 vs. want, 1-<2yrs p<0.001 vs. want, 2-<5 yrs
Want, not sure when	0.10	0.07, 0.14	p<0.001 vs. want, <1 yr p=0.001 vs. want, 1-<2yrs p=0.3 vs. want, 2-<5 yrs p=0.003 vs. want, 5+ years
Don’t want	0.02	0.02, 0.03	p<0.001 vs. want, <1 yr p<0.001 vs. want, 1-<2yrs p<0.001 vs. want, 2-<5 yrs p=0.3 vs. want, 5+ years p<0.001 vs. want, not sure when
*Importance:* How important is it to you to avoid becoming pregnant now? (N=8,920)^2^			
Not important at all	0.25	0.21, 0.29	ref
Somewhat unimportant	0.28	0.19, 0.38	p=0.5 vs. not important at all
Neither important nor unimportant	0.15	0.11, 0.19	p=0.001 vs. not important at all p=0.003 vs. somewhat unimportant
Somewhat important	0.14	0.11, 0.17	p<0.001 vs. not important at all p=0.001 vs. somewhat unimportant p=0.8 vs. neither important nor unimportant
Very important	0.05	0.04, 0.05	p<0.001 vs. not important at all p<0.001 vs. somewhat unimportant p<0.001 vs. neither important nor unimportant p<0.001 vs. somewhat important
*Wantedness:* Overall, thinking about the next year, do you want to become pregnant? (N=4,680)^3^			
Yes	0.35	0.29, 0.42	ref
Unsure, OK either way, don’t care	0.16	0.11, 0.21	p<0.001 vs. yes
No	0.05	0.04, 0.07	p<0.001 vs. yes p<0.001 vs. unsure, OK either way, don’t care

^1^Measured in Alabama (AL), Arizona (AZ), Iowa (IA), New Jersey (NJ), Ohio (OH), South Carolina (SC) and Wisconsin (WI) at wave 1 (missing in Delaware [DE] and Maryland [MD])

^2^Measured in all states at wave 1

^3^Measured in AL, IA, OH, and SC at wave 1 (missing in AZ, DE, MD, NJ, and WI)

## Discussion

In this analysis of state-representative data of reproductive-aged females across nine diverse US states, different prospective approaches to measuring fertility desires yielded different responses when administered to the same participants. Inconsistencies in which measure is used likely contribute to variability in results across studies.

The four fertility desire measurement approaches we assessed – DAP scores, Timing, Importance, and Wantedness – differ across features, including the number of questions/items, granularity of response categories, timeframe for pregnancy or childbearing, the directionality of assessment (for pregnancy vs. avoiding pregnancy) and, critically, the aspect of fertility desires being assessed. Our analysis highlights areas of trade-offs among these features and considerations when comparing Timing, Importance, and Wantedness to the DAP scale.

For instance, the approaches differ in degree of measurement information provided across fertility desire levels. The first category of Timing (want [a child], <1 year) and Wantedness (yes, want a pregnancy in the next year) largely captured individuals with the lowest DAP scores, and high percentages (35–40%) of participants in those categories experienced pregnancy. However, these approaches did not serve to differentiate among individuals with broad ranges of pregnancy preferences in the mid- and higher ranges of DAP scale scores. While the last category of Timing (don’t want), Importance (very important, avoiding pregnancy now), and Wantedness (no, do not want a pregnancy in the next year) captured the majority of people with high DAP scores (akin to high sensitivity), these categories also captured many individuals with mid-range DAP scores and some with low DAP scores (akin to low positive predictive value). In other words, approaches asking about wanting pregnancy capture many of those most open to pregnancy but do not differentiate individuals with a very broad range of pregnancy preferences among those who do not explicitly desire pregnancy, which is most people.

While responses to the extreme categories on the Timing question (want <1 year or want 1-<2 years, want >5 years or don’t want) correlated well with DAP scores, participants endorsing Timing’s middle response categories included those with a broad range of DAP scores. This finding is perhaps not surprising given the question’s focus on distinguishing participants’ desired *timing* of childbearing, vs. the DAP scale’s focus on capturing *multiple considerations* about childbearing within a one-year timeframe. Interestingly, mean DAP scores increased with increasing desired delay in childbearing. This pattern suggests that people may respond to desired timing questions based, in part, on how they feel about childbearing currently: the less favorably people feel about childbearing soon, the further out they estimate they might want a child.

Turning to the Importance question, results illustrate that the middle three categories (“somewhat important,” “neither,” and “somewhat unimportant”) did little to distinguish current pregnancy preferences. Additionally, responses to the question diverged from expectations regarding incident pregnancy: there was little difference in pregnancy among those responding “not important at all” vs. “somewhat unimportant,” as well as among those responding “neither” vs. “somewhat unimportant.” These findings call into question the utility of Importance’s middle response options both for ordering individuals by pregnancy preference and for anticipating pregnancy.

Differences in response patterns across individual questions and the DAP scale reflect and confirm differing conceptualization of pregnancy preferences used in the DAP scale vs. questions focusing exclusively on wantedness (Timing and Wantedness). In contrast to questions about proactively wanting pregnancy or a child, the DAP scale was developed with the idea that many individuals do not hold explicit wants or desires regarding childbearing, particularly individuals for whom reproduction does not feel salient at a particular time in their lives or those for whom attaining socially normative conditions to proactively want a or another child feels out of reach (Arteaga et al., 2019; Bhrolcháin & Beaujouan, 2019; Rocca et al., 2019). In this study, participants in the youngest and oldest age groups, as well as multiparous and Hispanic participants, were most likely to be open to pregnancy (per the DAP scale) but not want a child in the next year, highlighting that openness to pregnancy without an explicit desire for pregnancy may be elevated in these groups.

Notably, scholars have different goals when selecting a fertility desire measurement approach, and acknowledging trade-offs of each is important. For example, approaches that optimize valid and precise measurement may be suboptimal for prediction; similarly, a question or instrument may be highly predictive of an outcome while not validly capturing the construct (Smits et al., 2018). The explicit goal in developing the DAP scale was to select items that met prespecified psychometric criteria and validly captured the full scope of factors comprising pregnancy preferences. Had the goal been to predict incident pregnancy, different items might have been selected (Smits et al., 2018). Optimizing measurement validity and precision also requires multiple items, which adds to participant burden and may make score interpretation more challenging. On the flip side, while single questions are more efficient, lack of measurement precision or evidence that these items accurately categorize respondents in terms of fertility preferences is a compelling drawback to their use.

This study has limitations. Not all measurement approaches were included across all Surveys of Women states and waves, leading to slight differences in analytic samples that may affect comparability. For the Wantedness question, question wording varied by state, as did response options, which required collapsing “middle” categories for analysis. Pregnancy was self-reported, which likely resulted in underreporting, particularly of pregnancies that were less desired or ended in abortion (Desai et al., 2021). We therefore likely underestimate the predicted probability of pregnancy among those who report low fertility desires. Finally, the nine included states were not selected at random, and survey administration was staggered between 2017–2023; results therefore cannot be generalized to all reproductive-aged females in the US nor to any particular year. These limitations are balanced by important strengths, including use of a large, longitudinal dataset representative of reproductive-aged females across diverse US states.

Overall, our results suggest that research using different fertility desires measures is likely to yield different conclusions. Selection of an appropriate measurement approach will depend on the importance of measurement validity and precision, the purpose of measurement, ease of interpretation of response meaning, and tolerance of participant burden.

## Data Availability

The Surveys of Women data were made available to the research team through a Data Use Agreement with NORC, the custodian of the data. As such the research team is not at liberty to distribute data.

## Code Availability

Code will be made available upon request to the authors.
